# Supplementary material for: Independent rediploidization masks shared whole genome duplication in the sturgeon-paddlefish ancestor
Source: Nat Commun. 2023 May 19;14:2879. doi: 10.1038/s41467-023-38714-z (PMC10199039; doi:10.1038/s41467-023-38714-z)
Supplement: Supplementary file 1 — Supplementary Information [file 41467_2023_38714_MOESM1_ESM.pdf]

Supplementary Information for:

Independent rediploidization masks shared whole genome duplication in the sturgeon-paddlefish ancestor

Anthony K. Redmond[1], Dearbhaile Casey[1], Manu Kumar Gundappa[2], Daniel J. Macqueen[2], & Aoife McLysaght[1*]

[1]Smurfit Institute of Genetics, Trinity College Dublin, Dublin, Ireland

[2]The Roslin Institute and Royal (Dick) School of Veterinary Studies, University of Edinburgh, Edinburgh, UK

*aoife.mclysaght@tcd.ie

File includes **Supplementary Note 1** and **Supplementary Figures 1-10.**

<u>Supplementary Note 1:</u> ***Incomplete lineage sorting and introgression/admixture are not plausible alternatives to asynchronous rediploidization after WGD***

Although our detailed phylogenetic investigation of the sturgeon-paddlefish ohnolog pair subtree topology distribution clearly indicates that phylogenetic error is a very unlikely source for the asynchronous rediploidization signal that we observe, other biological phenomena are also known to produce conflicting topologies across gene trees. Key examples of this are incomplete lineage sorting, where ancestral allele diversity is differentially retained after subsequent speciation events such that the correct gene tree topology is discordant with the speciation history, and introgression and admixture, where there is genetic exchange after speciation resulting from hybridisation[1].

Hybridisation, which can lead to introgression and/or admixture, has been noted within and between sturgeons and paddlefish[2,3], meaning that genetic exchange of this type, particular shortly after their divergence, warrants serious consideration when examining the evolutionary history of their genomes. Despite this, we suggest that it is relatively unlikely to produce the data patterns we observe compared to asynchronous rediploidization. Firstly, the simplest hybridisation scenarios that result in a tree topology change will produce strongly supported 'Other' tree topologies (Supplementary Fig. 10), which are few in our data (Fig. 2C). In addition, the simplest scenarios that can generate the opposite topology (PreSpec to PostSpec and vice versa) requires parallel exchange between the two duplicate regions of each species (Supplementary Fig. 10). This seems unlikely to occur and not also be followed by any further exchange. In addition, if introgression or admixture, and not shared ancestral WGD, is the source of PreSpec topologies in our results, then our inferred WGD age should not be older than the speciation between sturgeon and paddlefish, as that would (without invoking much more complex scenarios) be the point of origin for all of the homologous chromosomes within and between species (Supplementary Fig. 10). This naturally also indicates that our Ks plot analyses are inconsistent with simple hybridisation scenarios as Ks values for PreSpec ohnolog pairs should also not be older than ortholog pair Ks values. Meanwhile, if PreSpec and 'Other' trees derive from hybridisation then they arguably should have lower Ks values than PostSpec (as they would derive from hybridisation disrupting an existing PostSpec history). Instead the Ks pattern we observe is more consistent with the distinct ohnolog divergence times as predicted under asynchronous rediploidization (Fig. 4). Perhaps most

pertinently, asynchronous rediploidization acts, in a sense, as a far simpler version of the same kind of genetic exchange. In this scenario homologous recombination between closely related chromosomes occurring at meiosis within the same species generates the observed pattern.

In the case of incomplete lineage sorting the simplest possible topology-altering scenarios for a duplicated gene tree i) should lead to strongly supported ‘Other’ trees, and ii) are more complex than those under a single copy gene tree topology (Supplementary Fig. 10). In order to produce a scenario where the opposing topology (either PreSpec or PostSpec depending on whether WGD is shared) is recovered, as is the case under asynchronous rediploidization, the lineage sorting history must be even more complex (Supplementary Fig. 10). This means that a WGD gene tree topology impacted by incomplete lineage sorting would most parsimoniously produce strongly supported ‘Other’ tree topologies as the primary alternative topology (or perhaps even the majority topology). Thus, incomplete lineage sorting predicts a gene tree distribution distinct from that of asynchronous lineage-specific rediploidization, and in the present case does not provide a parsimonious solution to the sturgeon-paddlefish ohnolog pair gene trees distribution we observe (Fig. 2C). Furthermore, we are unaware of any reason for a common incomplete lineage sorting scenario to be shared consistently along large doubly conserved syntenic genomic regions as is implied by the consistent recovery of PreSpec or PostSpec ohnologs in this manner (Fig. 3), as the allele history of different genes will usually be independent. Lastly, the Ks distribution we observe for the different topology categories is consistent with the gene tree topologies from which ohnolog pairs are derived (Fig. 4). If incomplete lineage sorting were the source of the disparate gene tree topologies then ohnolog pair Ks values would be scrambled by this relative to the distribution we observe, and ‘Other’ topologies should not occupy clearly distinct Ks distributions from PreSpec and PostSpec.

While incomplete lineage sorting and hybridisation may have played a role in acipenseriform genome evolution, they do not explain the gene tree, synteny, and Ks patterns we observe in our analysis of the sturgeon-paddlefish WGD. Beyond the present sturgeon-paddlefish case, it is clear that incomplete lineage sorting and introgression/admixture do not present the same challenges to WGD analyses of duplicated genes as they do to species tree inference studies on single copy genes (Supplementary Fig. 10). ILS and hybridisation are far less parsimonious explanations

for WGD evolutionary history patterns that can otherwise be elegantly ascribed to asynchronous, lineage-specific rediploidization.

1. Hibbins, M. S. & Hahn, M. W. Phylogenomic approaches to detecting and characterizing introgression. *Genetics* **220**, iyab173 (2022).
2. Havelka, M., Kašpar, V., Hulák, M. & Flajšhans, M. Sturgeon genetics and cytogenetics: a review related to ploidy levels and interspecific hybridization. *Folia Zool.* **60**, 93–103 (2011).
3. Káldy, J. *et al.* Hybridization of Russian Sturgeon (Acipenser gueldenstaedtii, Brandt and Ratzeberg, 1833) and American Paddlefish (Polyodon spathula, Walbaum 1792) and Evaluation of Their Progeny. *Genes* **11**, 753 (2020).

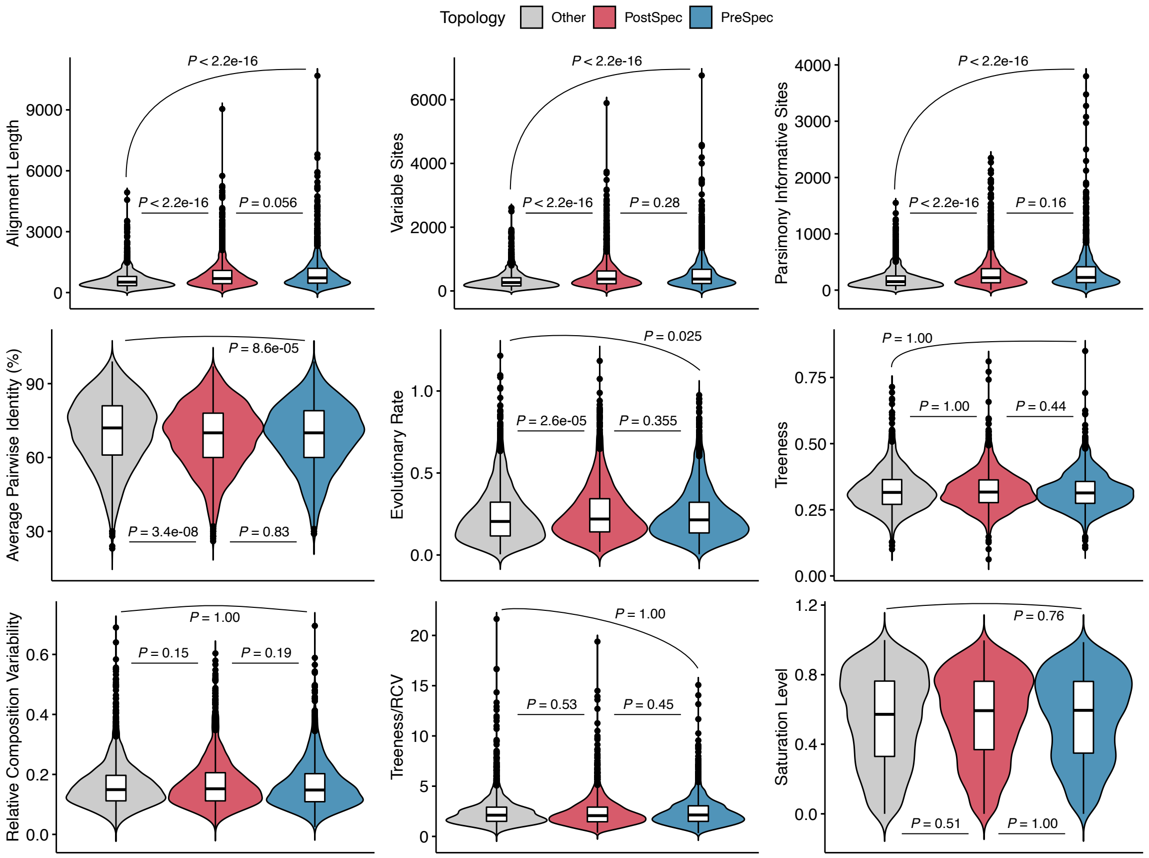

Supplementary Figure 1. Full violin and boxplots plots, as well as p-values for summary statistics comparing the three topology categories reported in Figure 2E. Boxplots are formed such that the middle value is a median (50% quantile), while the lower and upper hinges represent the 25% and 75% quantiles. Whiskers extend to the minima and maxima or extend to 1.5 times the interquartile range beyond the box (Tukey style) if outliers, drawn as individual data points, are present. Ohnolog pair PHOGs are split into Other (n=1917), PostSpec (n=2074), and PreSpec (n=1448) topology categories. P-values are derived from a two-sided Wilcox-test with Bonferroni correction in R and are shown for each comparison (exact values could not be computed due to ties in all comparisons). Source data are provided as a Source Data file.

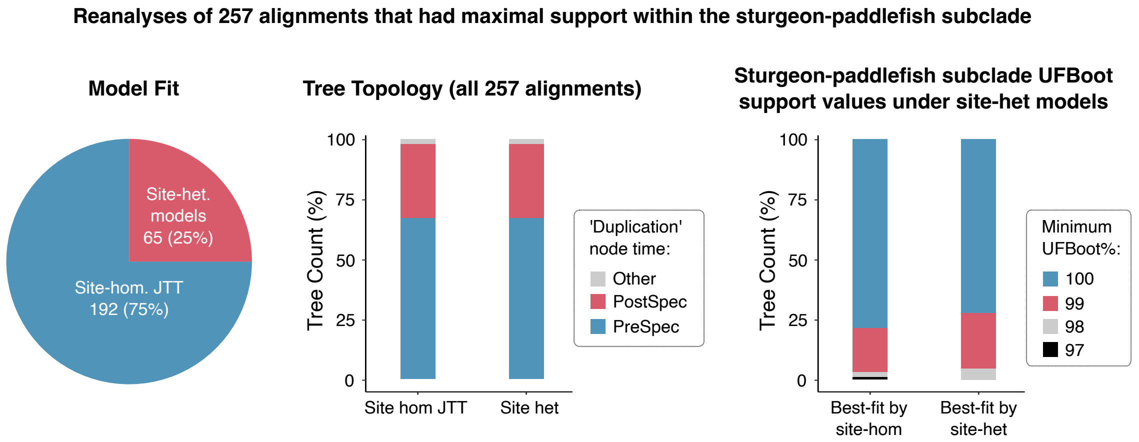

Supplementary Figure 2. Summary statistics for reanalyses with site-heterogeneous mixture models of the 257 alignments for which maximal support (UFBoot=100%) was recovered for the two branches in the sturgeon-paddlefish subclade. The proportion of alignments better fit by site-heterogeneous models as compared to the site-homogeneous JTT model are shown in a pie chart on the left. The frequency at which each of the three topology categories are recovered when always using the best-fitting site-heterogeneous models (including for the 75% of alignments where site-homogeneous models fit better) as compared to when all alignments are analysed with the site-homogeneous JTT model is shown in the centre. The impact that using site-heterogeneous models has on support values for all 257 alignments is shown on the right, with alignments better fit by site-homogeneous JTT or site-heterogeneous models shown separately. Source data are provided as a Source Data file.

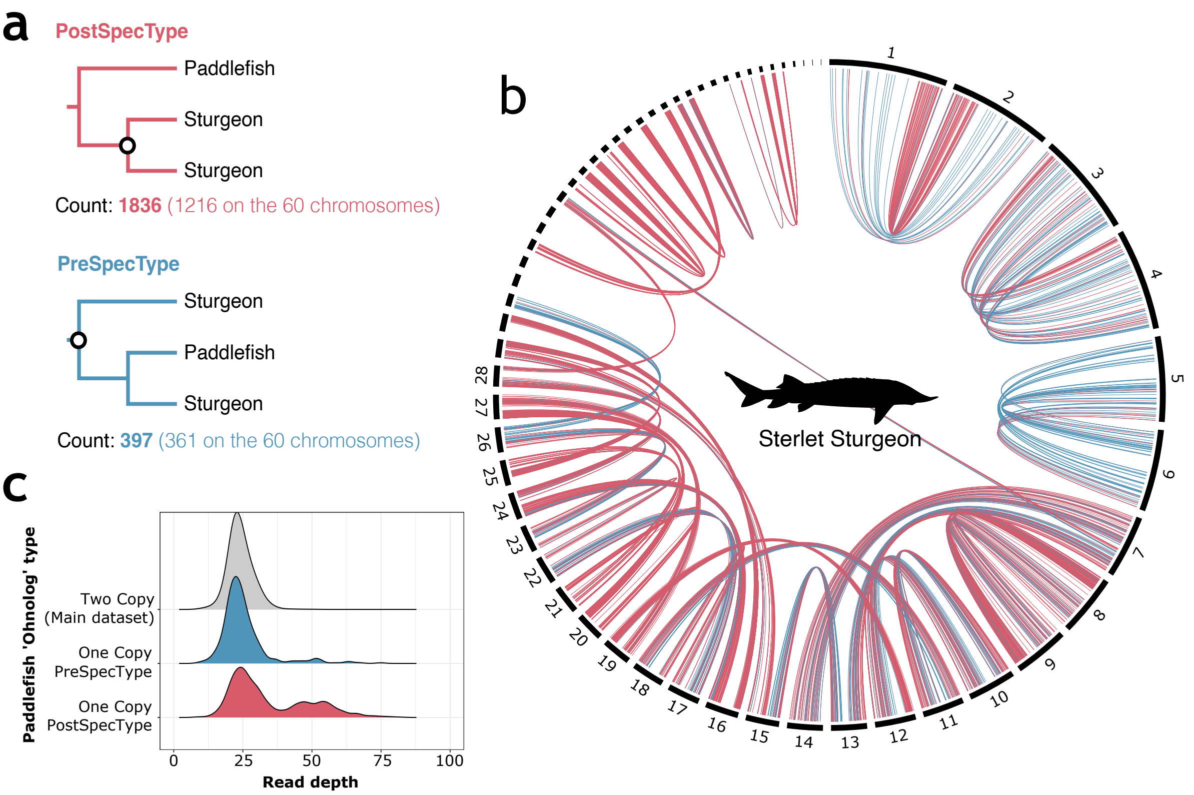

Supplementary Figure 3. Analyses of 2:1 sturgeon-paddlefish ohnolog pairs, where sturgeon retains both copies and the paddlefish genome contains only a single copy. (a) Frequency at which the PostSpec-type and PreSpec-type topologies are recovered in 2:1 ohnolog pair gene trees (unlike the main analysis we report paddlefish single copy sequences not present on the 60 chromosomes in the assembly, although only those on the 60 chromosomes are included parts b and c). (b) Circos plot showing synteny of PreSpec-type and PostSpec-type ohnolog pair links in the sturgeon genome as per Fig. 3A. (c) Ridgeline density plot of average genome sequencing read depth coverage for 2:1 sturgeon ohnolog pairs with either a PreSpec-type or PostSpec-type topology as compared to 2:2 ohnolog pairs (i.e., the main dataset). Source data are provided as a Source Data file. Raw alignments, gene trees, and gene tree parsing code are provided in figshare repository (https://doi.org/10.6084/m9.figshare.19762963.v1).

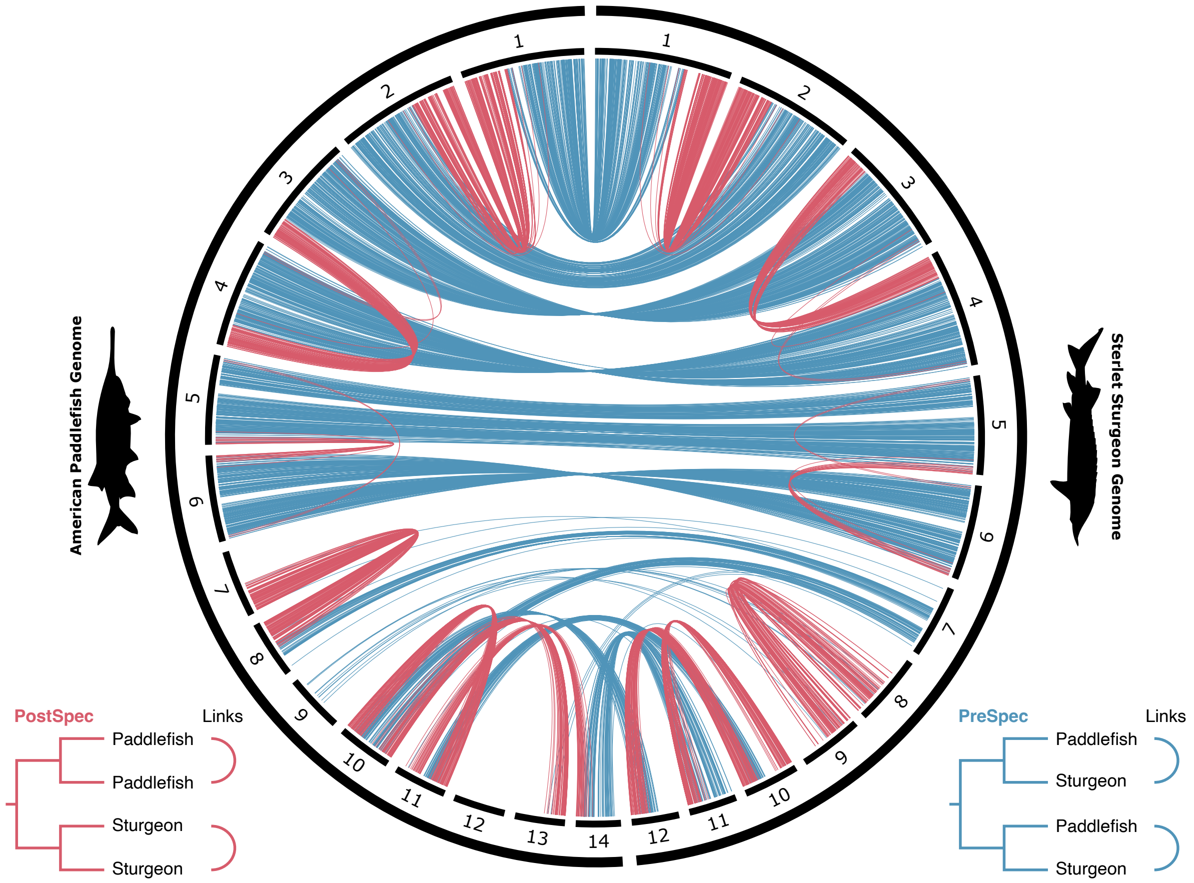

Supplementary Figure 4. Circos plot showing synteny of PreSpec and PostSpec ohnolog pairs links as per Fig. 3C, but for the macrochromosomes >40Mb only. Source data are provided as a Source Data file.

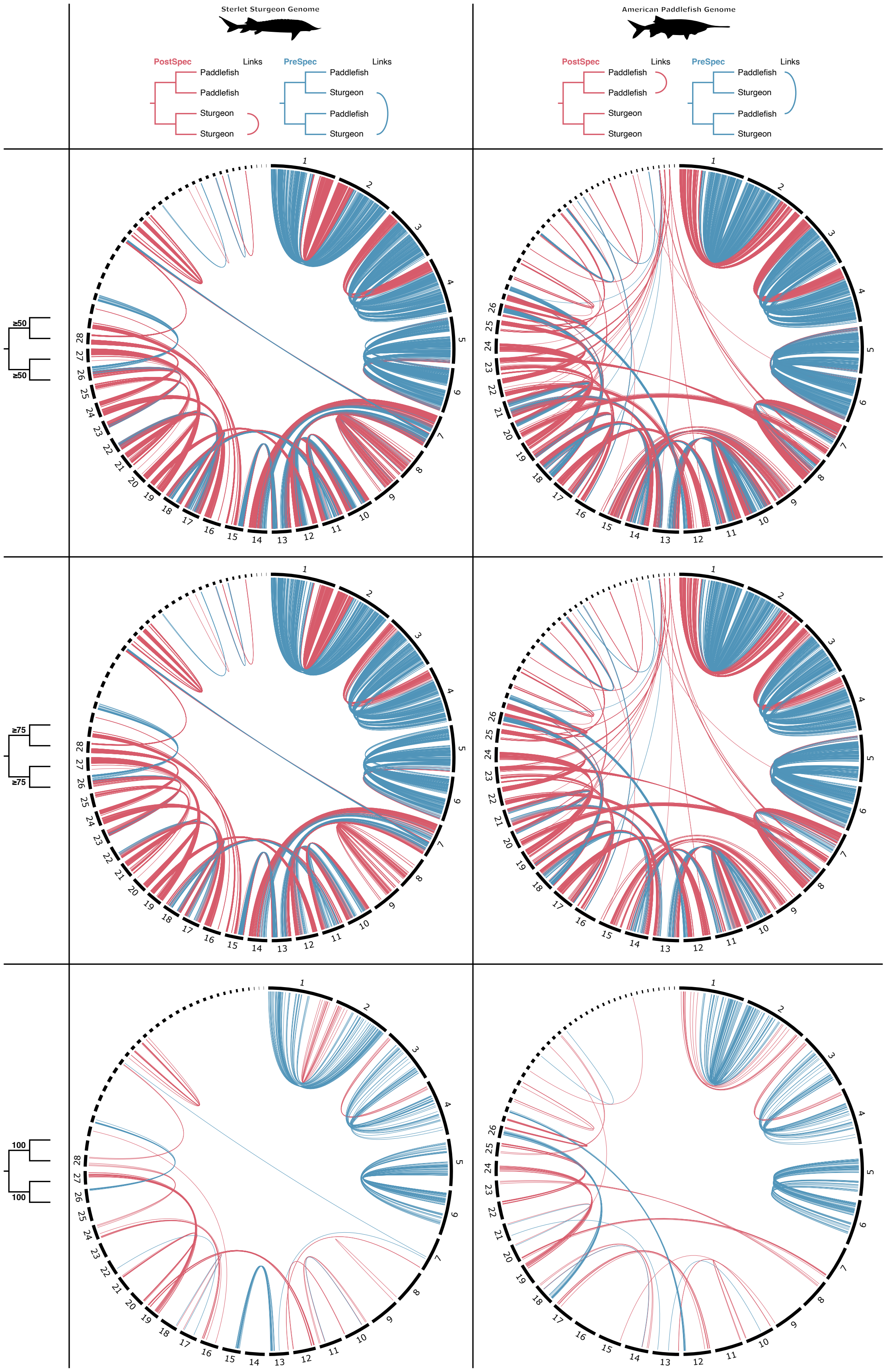

Supplementary Figure 5. Circos plot showing synteny of PreSpec and PostSpec ohnolog pairs along the sturgeon (left) and paddlefish (right) genomes as per Fig. 3A and Fig. 3B at increasingly stringent UFBoot cut-off% values from top to bottom (UFBoot ≥50%; UFBoot ≥75%; UFBoot =100%). Source data are provided as a Source Data file.

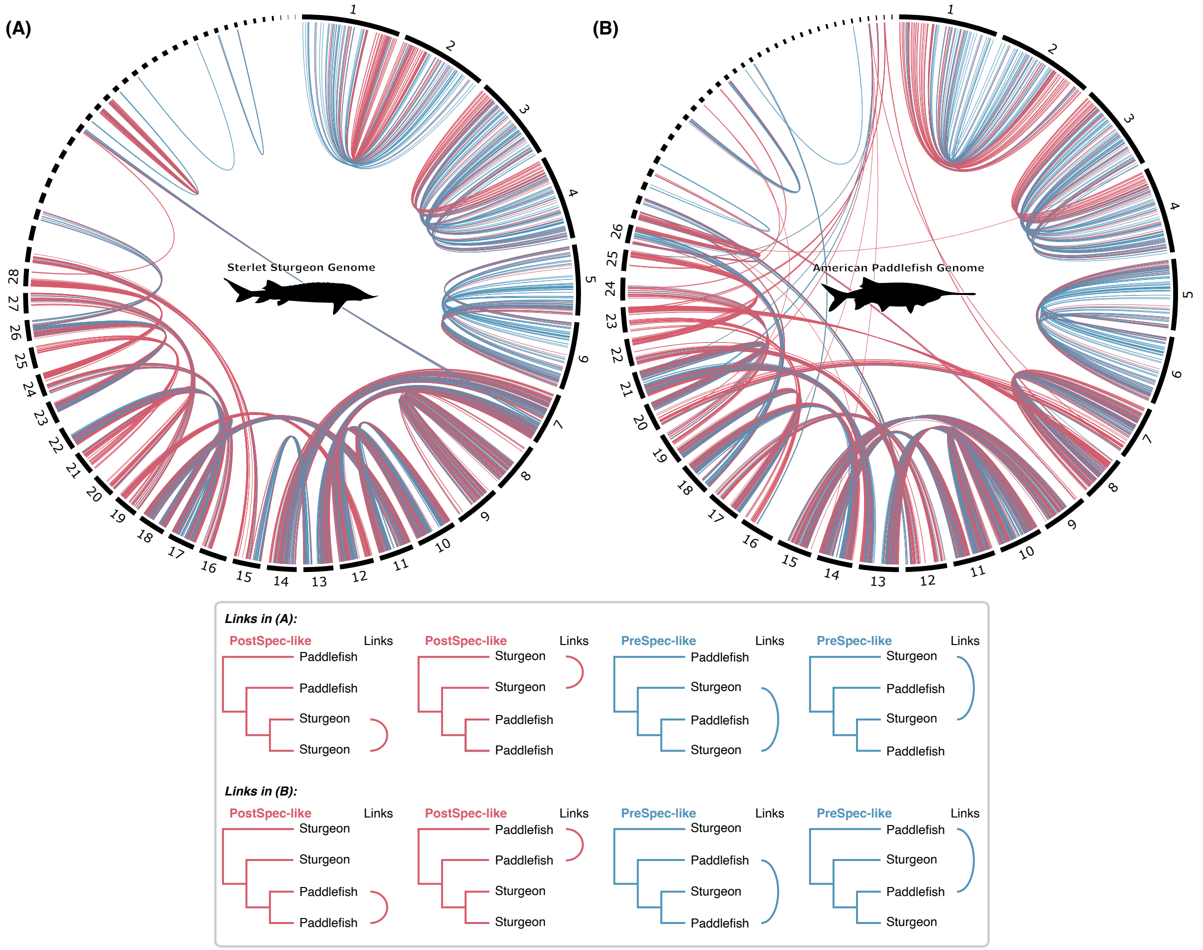

Supplementary Figure 6. Circos plots of synteny patterns of 'Other' topology 'PreSpec-like' and 'PostSpec-like' recovering ohnolog pairs in the (A) sturgeon and (B) paddlefish genomes. Other details as per Fig. 3A and Fig. 3B. Source data are provided as a Source Data file.

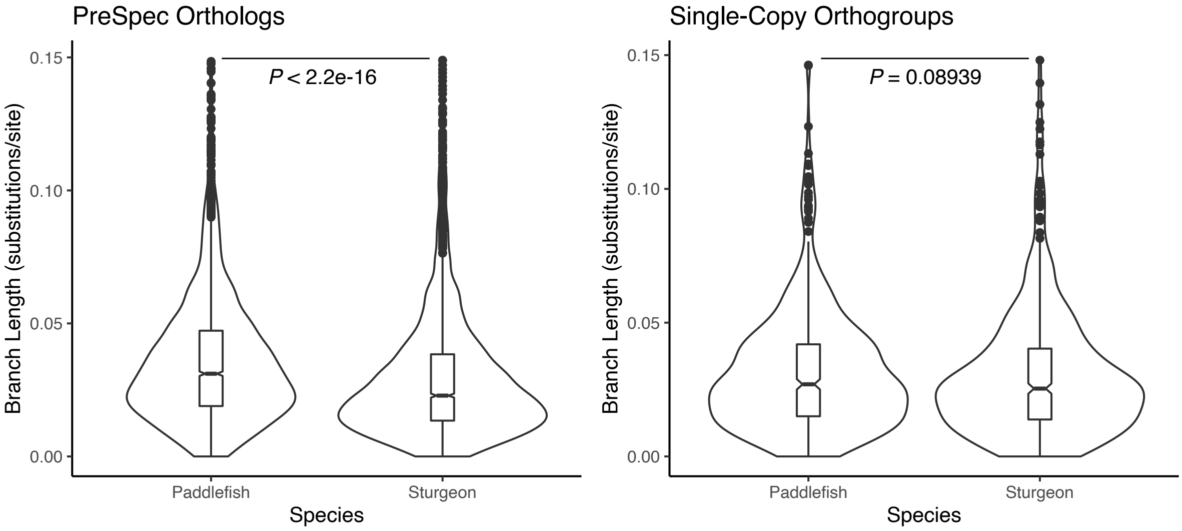

Supplementary Figure 7. Violin and boxplots plots, as well as p-values, for analyses comparing the branch length in substitutions per site (and hence inferred amino acid evolutionary rate) of sturgeon and paddlefish PreSpec Orthologs and Single-Copy Orthogroups. Boxplots are formed such that the middle value is the median (50% quantile), while the lower and upper hinges represent the 25% and 75% quantiles. Whiskers extend to the minima and maxima or extend to 1.5 times the interquartile range beyond the box (Tukey style) if outliers, drawn as individual data points, are present. Pairs where one branch length was ≥ 0.15 substitutions per site were excluded as extreme outliers from both visualisation and statistical comparison. n=2829 pairs for PreSpec Orthologs and n=478 pairs for Single-Copy Orthogroups. P-values are derived from a one-sided Wilcox-test in R resulting in $p < 2.2e-16$ for PreSpec Ortholog pairs indicating that branch lengths are significantly longer in paddlefish than sturgeon, while $p = 0.08939$ for Single-Copy Orthogroup pairs indicating no significant difference in branch lengths between the species for this dataset. Exact p-values could not be calculated due to ties. Source data are provided as a Source Data file. Raw alignments, gene trees, and gene tree parsing code are provided in figshare repository (https://doi.org/10.6084/m9.figshare.19762963.v1).

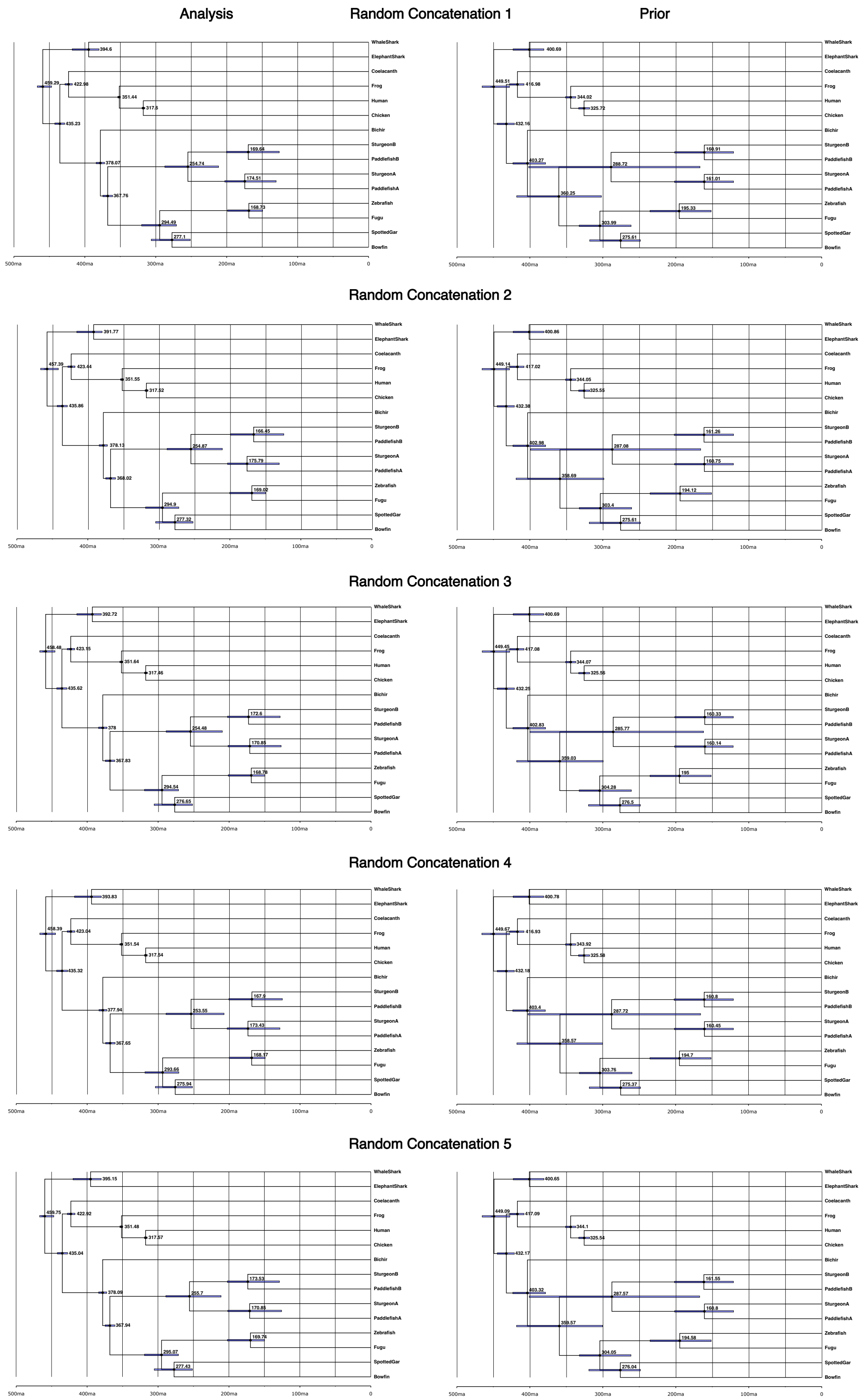

Supplementary Figure 8. Phylogenomic dating analyses with all fish calibrations as recovered from all 5 random concatenations with mean divergence dates and 95% credibility intervals (blue-violet bar) shown for each node. Runs under the prior are shown on the right. Source data are provided in figshare repository (https://doi.org/10.6084/m9.figshare.19762963.v1). Calibrations are in Supplementary Data 1.

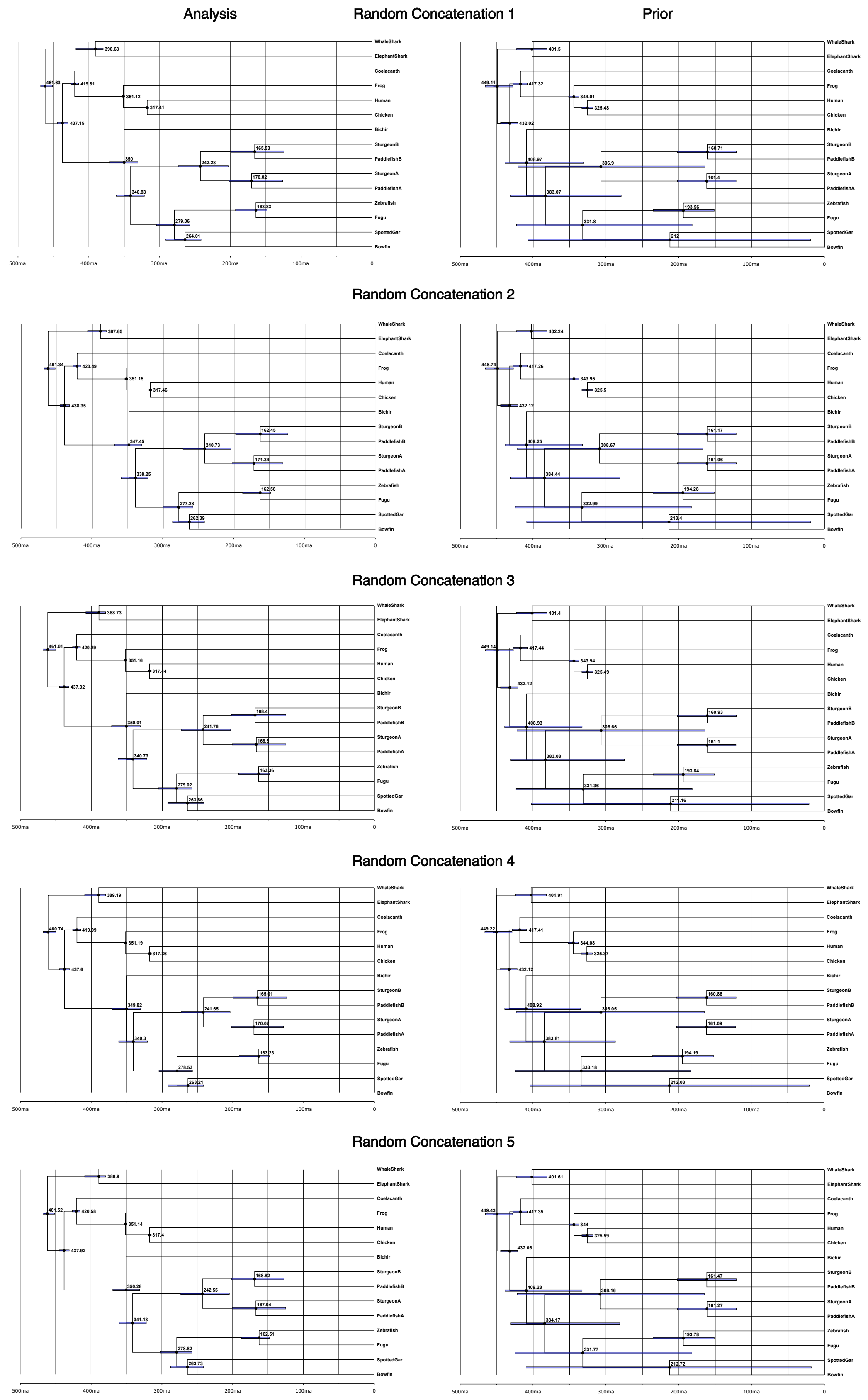

Supplementary **Figure 9.** Phylogenomic dating analyses with fewer fish calibrations as recovered from all 5 random concatenations with mean divergence dates and 95% credibility intervals (blue-violet bar) shown for each node. Runs under the prior are shown on the right. Source data are provided in figshare repository (https://doi.org/10.6084/m9.figshare.19762963.v1). Calibrations are in Supplementary Data 1.

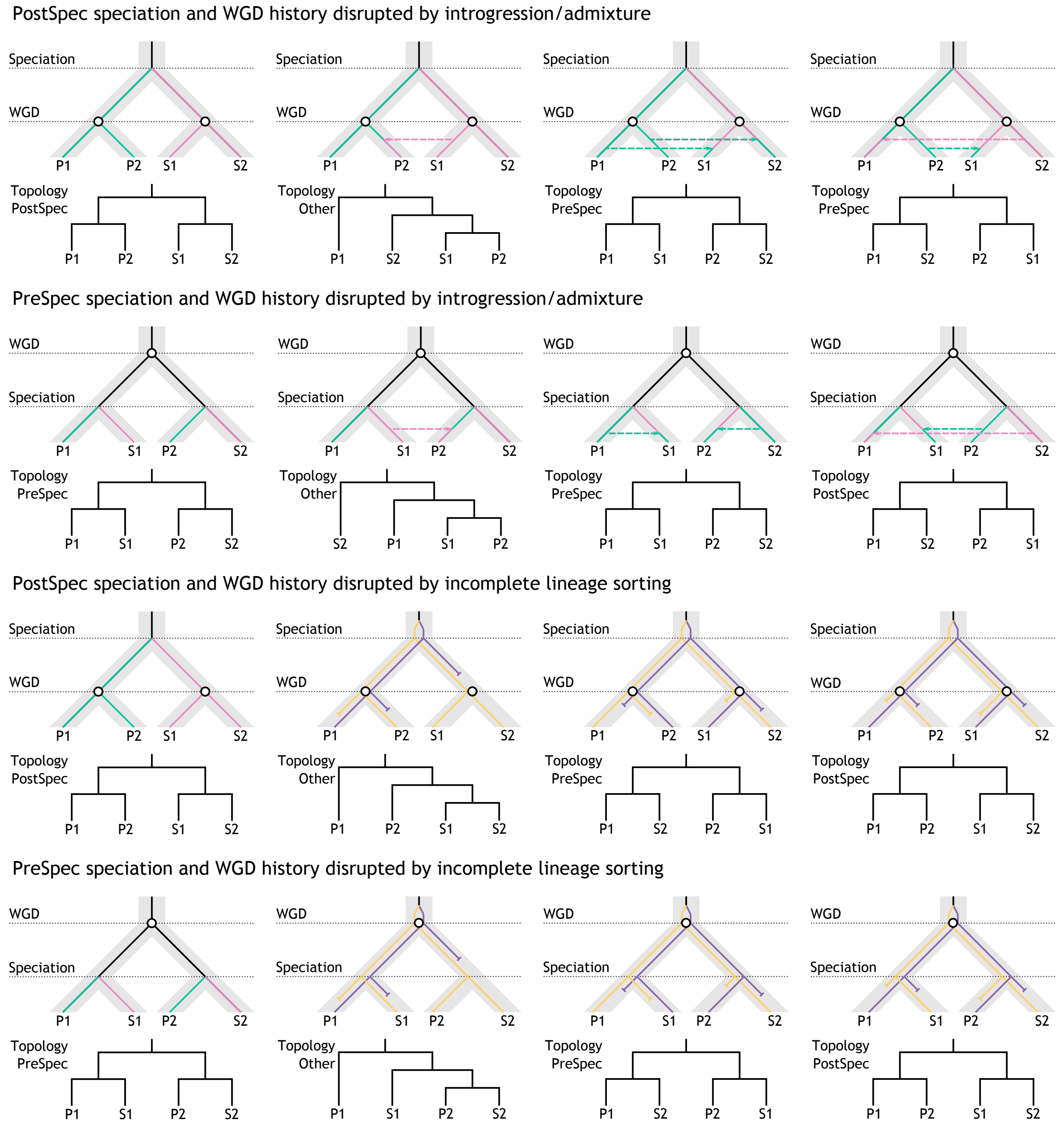

Supplementary Figure 10. Examples of the simplest phylogenetic scenarios under which incomplete lineage sorting and gene flow from hybridisation (introgression/admixture) might influence sturgeon(species S)-paddlefish (species P) ohnolog pair subtree topologies. The gene tree following an unperturbed speciation and WGD history is shown on the left of each row. Colours represent the speciation history (green=paddlefish [species P]; pink=sturgeon [species S]) of that gene for unperturbed histories (left column) and introgression/admixture scenarios (top two rows). The second, third, and fourth columns of the bottom two rows are instead coloured according to two alleles that diverged prior to speciation (yellow and purple). Asynchronous rediploidization is not considered, as otherwise all alternative scenarios simply add additional complexity. We instead consider two alternative scenarios of genome duplication (and immediate rediploidisation) occurring before (PreSpec) or after (PostSpec) the sturgeon-paddlefish species divergence.
