# Supplementary material for: Independent rediploidization masks shared whole genome duplication in the sturgeon-paddlefish ancestor
Source: Nat Commun. 2023 May 19;14:2879. doi: 10.1038/s41467-023-38714-z (PMC10199039; doi:10.1038/s41467-023-38714-z)
Supplement: Supplementary file 3 — Description of Additional Supplementary Files [file 41467_2023_38714_MOESM3_ESM.pdf]

Description of Additional Supplementary Files

File Name: **Supplementary Data 1**.
Description: This file contains Fossil calibrations used and the results of five random concatenations for phylogenomic dating of the sturgeon-paddlefish WGD.
