# Supplementary material for: Independent rediploidization masks shared whole genome duplication in the sturgeon-paddlefish ancestor
Source: Nat Commun. 2023 May 19;14:2879. doi: 10.1038/s41467-023-38714-z (PMC10199039; doi:10.1038/s41467-023-38714-z)
Supplement: Supplementary file 5 — Reporting Summary [file 41467_2023_38714_MOESM5_ESM.pdf]

nature portfolio

Corresponding author(s): Aoife McLysaght

Last updated by author(s): Apr 29, 2023

Reporting Summary

Nature Portfolio wishes to improve the reproducibility of the work that we publish. This form provides structure for consistency and transparency in reporting. For further information on Nature Portfolio policies, see our Editorial Policies and the Editorial Policy Checklist.

Statistics

For all statistical analyses, confirm that the following items are present in the figure legend, table legend, main text, or Methods section.

n/a	Confirmed	
☐	☒	The exact sample size (*n*) for each experimental group/condition, given as a discrete number and unit of measurement
☒	☐	A statement on whether measurements were taken from distinct samples or whether the same sample was measured repeatedly
☐	☒	The statistical test(s) used AND whether they are one- or two-sided<br>*Only common tests should be described solely by name; describe more complex techniques in the Methods section.*
☒	☐	A description of all covariates tested
☐	☒	A description of any assumptions or corrections, such as tests of normality and adjustment for multiple comparisons
☐	☒	A full description of the statistical parameters including central tendency (e.g. means) or other basic estimates (e.g. regression coefficient) AND variation (e.g. standard deviation) or associated estimates of uncertainty (e.g. confidence intervals)
☐	☒	For null hypothesis testing, the test statistic (e.g. *F*, *t*, *r*) with confidence intervals, effect sizes, degrees of freedom and *P* value noted<br>*Give P values as exact values whenever suitable.*
☐	☒	For Bayesian analysis, information on the choice of priors and Markov chain Monte Carlo settings
☒	☐	For hierarchical and complex designs, identification of the appropriate level for tests and full reporting of outcomes
☒	☐	Estimates of effect sizes (e.g. Cohen's *d*, Pearson's *r*), indicating how they were calculated

Our web collection on statistics for biologists contains articles on many of the points above.

Software and code

Policy information about availability of computer code

Data collection	no specific software was used for data collection.
Data analysis	No commercial code was used.<br>Open source code: Orthofinder (v. 2.5.4); MAFFT (v7.487); trimAl (v1.4.rev15); BMGE (v. 1.12); IQ-tree (v. 2.1.4-beta COVID-edition); ETE (v3); Hmmer 552 package (v3.1b2); circos-0.69-9; Phylobayes (v4.1c); wgd; PhyKIT (v. 1.11.3); Bowtie (v 2.4.2); SAMtools (v 1.16.1); mosdepth (v 0.3.3); R (v. 4.1.2 [2021-11-01]).<br>Custom code: All ETE3 python scripts are available on figshare (10.6084/m9.figshare.19762963).<br>Code availability statement in manuscript: All custom ETE3-based gene tree parsing python scripts are available on figshare (10.6084/m9.figshare.19762963).<br>All custom ETE3-based gene tree parsing python scripts are available on figshare (10.6084/m9.figshare.19762963).

For manuscripts utilizing custom algorithms or software that are central to the research but not yet described in published literature, software must be made available to editors and reviewers. We strongly encourage code deposition in a community repository (e.g. GitHub). See the Nature Portfolio guidelines for submitting code & software for further information.

Data

Policy information about availability of data

All manuscripts must include a data availability statement. This statement should provide the following information, where applicable:

- Accession codes, unique identifiers, or web links for publicly available datasets
- A description of any restrictions on data availability
- For clinical datasets or third party data, please ensure that the statement adheres to our policy

The alignments, gene trees, random concatenation supermatrices, and phylogenomic dating chronograms generated in this study are provided on figshare (10.6084/m9.figshare.19762963). The phylogenomic dating node calibrations and inferred ages generated in this study are provided in Supplementary Data 1. The inferred topology categories, AU-test results, UFBoot cut-offs, off-target clade recovery, alignment, modelling and gene tree statistics, synteny data, Ks values, read depth coverage across paddlefish ohnologs, and ortholog branch length data generated in this study are provided in the Source Data file. The DNA-sequencing read data used to assess possible assembly collapse of ohnologous regions in the paddlefish genome in this study are available in the CNGBdb database under accession codes CNX0162203-5 (from project CNP0000867 available at https://db.cngb.org/search/project/CNP0000867/).

Human research participants

Policy information about studies involving human research participants and Sex and Gender in Research.

Reporting on sex and gender	n/a
Population characteristics	n/a
Recruitment	n/a
Ethics oversight	n/a

Note that full information on the approval of the study protocol must also be provided in the manuscript.

Field-specific reporting

Please select the one below that is the best fit for your research. If you are not sure, read the appropriate sections before making your selection.

☒ Life sciences ☐ Behavioural & social sciences ☐ Ecological, evolutionary & environmental sciences

For a reference copy of the document with all sections, see nature.com/documents/nr-reporting-summary-flat.pdf

Life sciences study design

All studies must disclose on these points even when the disclosure is negative.

Sample size	Sample size was neither predetermined nor was a sample size calculation performed. The species used were specifically relevant to the problem at hand (outgroups were sampled in a balanced manner across the species tree branches), while all protein coding genes from each species were considered in our analyses. In all cases the maximum number of pertinent ohnolog pairs/genes/gene family trees etc. were used.
Data exclusions	A very small number of data points were excluded from our Ks analyses and from our ortholog branch length analyses on the basis that they represent very extreme outliers. Specifically this included Ks values over 0.3 and branch lengths over 0.15 substitutions per site.
Replication	Our study is computational in nature. The data and custom code (including appropriately formatted input data files) required for replication have been provided as part of the figshare repository (10.6084/m9.figshare.19762963), Supplementary Data 1 and Source Data.
Randomization	We generated five distinct datasets by randomly assigning ohnologs from a pair as the 'A' or 'B' copy prior to concatenation of all 81 existing gene family multiple sequence alignments. This avoid bias from a single arbitrary concatenation, while also permitting assessment of how robust results are to variations in ohnolog concatenations [reproduced here from the manuscript methods section focused on using phylogenomics to identify a lower bound date for the sturgeon-paddlefish WGD].
Blinding	Traditional blinding is not usually applied to this type of study. The species used were specifically relevant to the problem at hand (outgroups were sampled in a balanced manner across the species tree branches), while all protein coding genes from each species were considered in our analyses. A core basis of our study is assignment of gene trees and ohnolog pairs to distinct rediploidization times (e.g. PostSpec or PreSpec).

Reporting for specific materials, systems and methods

We require information from authors about some types of materials, experimental systems and methods used in many studies. Here, indicate whether each material, system or method listed is relevant to your study. If you are not sure if a list item applies to your research, read the appropriate section before selecting a response.

Materials & experimental systems

n/a	Involved in the study
☒	☐ Antibodies
☒	☐ Eukaryotic cell lines
☒	☐ Palaeontology and archaeology
☒	☐ Animals and other organisms
☒	☐ Clinical data
☒	☐ Dual use research of concern

Methods

n/a	Involved in the study
☒	☐ ChIP-seq
☒	☐ Flow cytometry
☒	☐ MRI-based neuroimaging
